# Effect of vaporized hydrogen peroxide reprocessing on N95 respirators

**DOI:** 10.1017/ice.2020.371

**Published:** 2020-07-24

**Authors:** Elena Beam, Jeffrey C. Nesbitt, Matthew D. Austin, Kannan Ramar

**Affiliations:** 1 Division of Infectious Diseases, Mayo Clinic, Rochester, Minnesota; 2 Occupational Safety, Rochester, Minnesota; 3 Division of Pulmonary and Critical Care Medicine, Center for Sleep Medicine, Mayo Clinic, Rochester, Minnesota

*To the Editor—*The high demand for personal protective equipment (PPE) during the coronavirus disease 2019 (COVID-19) pandemic has required reprocessing and reuse of N95 respirators to mitigate shortages. Data on the impacts of reprocessing and reuse on the physical integrity and continued performance of these PPE are sparse.

Our facility uses vaporized hydrogen peroxide (VHP) according to the Duke method as a strategy to reprocess respirators for reuse.^1^ We conducted repeated quantitative fit testing of N95 respirators (model 3M 1870+, 3M, Maplewood, MN) by measuring the amount of leakage into the facepiece. We sought to better understand the impact of VHP reprocessing on reuse and extended reuse of respirators as well as the effect on the tight fit of the respirator in 2 experiments.

In our first experiment, 5 masks that were reprocessed with VHP were compared to 5 masks that were not treated with VHP. Quantitative fit testing was conducted using TSI PortaCount according to the Occupational Safety and Health Administration (OSHA) quantitative fit testing protocol. Safety staff conducted repeat use and fit tests in 10 cycles as follows: (1) Inspect the respirator. (2) Adjust and don the respirator. (3) Perform a quantitative respirator fit test. (4) Doff the respirator. And (5) flatten the respirator. Any observable failures and fit-test failures were documented after each cycle (Table 1). We found no fit-testing failure among the respirators treated with VHP, while the 5 control respirators had 4 total fit-test failures. Notably, 3 failures occurred after the ninth cycle of donning and doffing the respirators.

**Table 1.** Respirator Fit Test Cycle Results

Respirator No.	Fit Test Cycle									
	1	2	3	4	5	6	7	8	9	10
Control 1^a^	Pass	Pass	Pass	Pass	Pass	Pass	Pass	Pass	Pass	Pass
Control 2	Pass	Pass	Fail	Pass	Pass	Pass	Pass	Pass	Fail	Pass
Control 3	Pass	Pass	Pass	Pass	Pass	Pass	Pass	Pass	Pass	Pass
Control 4	Pass	Pass	Pass	Pass	Pass	Pass	Pass	Pass	Pass	Pass
Control 5	Pass	Pass	Pass	Pass	Pass	Pass	Pass	Pass	Fail	Fail
VHP 1^b^	Pass	Pass	Pass	Pass	Pass	Pass	Pass	Pass	Pass	Pass
VHP 2	Pass	Pass	Pass	Pass	Pass	Pass	Pass	Pass	Pass	Pass
VHP 3	Pass	Pass	Pass	Pass	Pass	Pass	Pass	Pass	Pass	Pass
VHP 4	Pass	Pass	Pass	Pass	Pass	Pass	Pass	Pass	Pass	Pass
VHP 5	Pass	Pass	Pass	Pass	Pass	Pass	Pass	Pass	Pass	Pass

a Control: 1870 respirator used without reprocessing.

b VHP: 1870 respiratory used with vaporized hydrogen reprocessing.

In our second experiment, to better understand the impact of VHP reprocessing on reuse and extended use (defined as repeat half-day use, or 4-hour shifts), we tested the same type of respirators (3M 1870) according to the following procedure: (1) The respirator was used for 4 hours. (2) The respirator was reprocessed with VHP. (3) The respirator was used for an additional 4 hours. (4) The respirator was reprocessed with VHP. And (5) a quantitative fit test was performed on the respirator. Respirators used in this experiment had already been used in hospital service by a single user for a clinical purpose. The duration of use was unknown.

All 5 respirators successfully passed the quantitative fit testing. The results from both experiments suggested that reprocessing with VHP allows for reuse and extended use of respirators. Our study has several limitations. These results might not be generalizable to other contexts. The quality and integrity of respirator used may differ by brand and style. Variability in the results may have resulted from the use of quantitative versus qualitative fit-testing techniques, which may be more prone to error due to the subjective nature of the test. Our evaluations were based on conclusions from quantitative testing.^2.3^ Finally, fit-testing expertise among the staff who volunteered to run the experiment varied; however, we expect our data to be reliable because the same staff performed multiple tests. Finally, we were not able to capture the duration of clinical use of the respirators in the hospital prior to the start of our second experiment, and we were unable to define the overall “use” in the project given this limitation.

Limited evidence is available regarding the use of VHP with extended reuse of N95 respirators.^4,5^ Similar to other studies, we found no detrimental effect of VHP processing on the ability of N95 respirators to pass fit testing. Our results suggest that VHP does not affect limited reuse and extended use of 3M-1870 respirators in the context of maintenance fit testing with repeated donning and doffing.
